# Exit, O Sodium!

**DOI:** 10.1093/function/zqae018

**Published:** 2024-04-08

**Authors:** Sergej Pirkmajer, Alexander V Chibalin

**Affiliations:** Institute of Pathophysiology, Faculty of Medicine, University of Ljubljana, Ljubljana 1000, Slovenia; Karolinska Institutet, Department of Molecular Medicine and Surgery, Integrative Physiology, Stockholm 17177, Sweden; National Research Tomsk State University, Tomsk 634050, Russia

Since Ole H. Peterson’s 2021 editorial “*When a Discovery Is a Rediscovery: Do We Know the History of Our Own Subject?*” highlighted the lack of rigor in reporting historical background in research papers,^1^ the importance of proper referencing and understanding the history of our research fields has been repeatedly emphasized^2,3^ in *Function*. Historical distance, untimely discovery in an unreceptive environment, inaccessibility of original literature, language barriers, and/or citation bias can all contribute to obscuring readily available facts,^1–^ ^5^ ultimately leading to their oblivion. Surprisingly, however, sometimes the errors are being propagated even though are visible to all.

The origins of scientific terms in biomedicine, some of which go back to the antiquity, are no less prone to being forgotten than original discoveries. For instance, it is difficult to resolve what holiness, mice, and fish have in common with the sacral bone (*os sacrum*—the “holy” bone),^6^ muscle (*musculus*—a little mouse),^7^ or the big toe—*hallux* (supposedly originating from *hal(l)ex, hal(l)ec*, or *al(l)ec*—a fish sauce in ancient Rome),^7^ respectively. Given the uncertain origins of these terms, contradictory interpretations, such as that of the name of the saphenous vein, which could mean both the obvious and the hidden vein,^8^ are hardly surprising. However, it is less clear why recent, well-documented scientific coinages should be misinterpreted and almost completely replaced by wrong forms.

A low intracellular concentration of Na^+^ and a high intracellular concentration of K^+^ is a fundamental characteristic of living cells. In animal cells, asymmetric ion distribution is maintained by an Na^+^-K^+^ pump,^9–^ ^12^ a P-type ATPase that catalyses the reaction: ATP + H_2_O + 3Na^+^_(in)_ + 2K^+^_(out)_ → ADP + P_i _+ 3Na^+^_(out)_ + 2K^+^_(in)_.^13,14^ In yeast and protists, an Na^+^-K^+^ pump does not exist, and it was long assumed that an H^+^ pump, a V-type ATPase, is primarily responsible for actively maintaining ion gradients.^15^ However, in 1991, a new putative P-type ATPase, which is now known to catalyse the reaction ATP + H_2_O + Na^+^_(in)_ → ADP + P_i _+ H^+^ + Na^+^_(out)_,^16–^ ^19^ was described in *Saccharomyces cerevisiae*.^15^ Clearly, the yeast also possessed a primary active Na^+^ extrusion system! In the classical tradition, the gene encoding the newly described ATPase was promptly named *ENA1* from *exitus natrii*,^15^ meaning the exit of sodium. Later, other related ATPases were described, and it is now evident that there is a family of Na^+^-extruding ATPases^20–^ ^22^ that transport Na^+^ in fungi,^15^ protists,^23^ and plants.^16^

The official gene names, which unambiguously denote a single, specific gene, are frequently abbreviations (eg, *GAPDH*) of the name of the encoded protein (eg, glyceraldehyde-3-phosphate dehydrogenase). However, in cases such as *ENA1*, the link between the name of the gene (*ENA1*) and the encoded protein (sodium/potassium exporting P-type ATPase 1) is not obvious. Indeed, the name of the gene *ENA1* does not make any intuitive sense unless the history of its discovery is known. While the acronym *ENA1* caught on quickly, the Latin phrase *exitus natrii*, from which it originated, was mentioned only occasionally,^24–^ ^26^ but was typically not referred to in research papers for nearly 20 yr^27–^ ^30^ after its discovery.^15^ In a reverse trend, the acronym is often explained in more recent sources, possibly to explain why a gene for a sodium P-type ATPase (a sodium pump) was called *ENA1*.

Interestingly, however, *exitus natrii* has been wrongly, but consistently, transmuted into *exitus natru* in sources ranging from research papers and reviews^20^,^31–^ ^44^ to student theses,^45–^ ^61^ books,^62,63^ encyclopedias (eg, *Na^+^-ATPase in Protozoan Parasites | Encyclopedia MDPI*), and even genome and other online databases (eg, *Saccharomyces Genome Database, BioCyc Pathway/Genome Database Collection, YeastPhenome.org, Alliance of Genome Resources*, and *Pathway Genome Databases of Agriculturaly Important Bacteria Sequenced by the USDA*). A simple Google search returns hundreds of hits for ^“^*exitus natru,^”^* but only a couple for “*exitus natrii*.” The use of *exitus natru* instead of *exitus natrii* is so pervasive that it even appears in abstracts of various research papers.^20,32,41^

*Exitus natru* is usually explained as meaning the exit of sodium, implying that *natru* is the genitive. However, *natrium*, the Neo-Latin for sodium, follows the second Latin declension (neuter):

**Table:** 

Nominative	*natrium*
Genitive	*natrii*
Dative	*natrio*
Accusative	*natrium*
Vocative	*natrium*
Ablative	*natrio*

In accordance with this convention, PubChem, an open chemistry database at the National Institutes of Health (NIH), uses *natrii* in the Latin names of over 300 sodium compounds and substances, while *natru* does not occur once in this context. Obviously, the exit of sodium should be translated into Latin as *exitus natrii* as written by the discoverers of *ENA*.^15^ The origin of *natru* in the scientific literature is therefore unclear. It is possible that it derives from the closely related classical term *nitrum*, meaning soda. *Nitrum* was derived from the ancient Greek νίτρον, whose genitive is νίτρου (pronounced nitru), which is similar to *natru*. However, *nitrum* is not a synonym for sodium, so such a usage would be difficult to justify. And even if it were, the correct form would be *exitus nitru* not *exitus natru*. On the other hand, the particular type of the italic font used in the original publication yields *ii*, which look almost exactly like *u*,^15^ suggesting that *natrii* may simply have been misread as *natru*.

In a further twist, *exitus natru* is sometimes explained as “exit sodium,”^31,34,38,41,45,47,49,50,57,58,63^ which would make *exitus* a verb (an imperative) and by extension *natru* a vocative (O sodium!). The latter translation would certainly have amused Winston Churchill, who, in his memoir *My Early Life*,^64^ wittily described the bewilderment at having to learn the vocative of the first Latin declension:

“Mensa, O table, is the vocative case,” he [ie, the Master] replied.

“But why O table?” I persisted in genuine curiosity.

“O table, you would use that in addressing a table, in invoking a table.” And then seeing he was not carrying me with him, “You would use it in speaking to a table.”

“But I never do,” I blurted out in honest amazement.

ChatGPT also seems to know Latin very well. When asked what *exitus natrii* means, it replies that “‘*Exitus natrii*’ translates to ‘exit of sodium’ in English.” In contrast, ChatGPT claims: “The phrase ‘*exitus natru*’ itself doesn’t seem to correspond directly to a known Latin expression or word.” When asked again about the meaning of the word *natru*, ChaptGPT insists: “As of my last knowledge update in January 2022, ‘*natru*’ doesn’t appear to be a recognized Latin word.”

Bioscientific and medical terminology is full of mythological, even somewhat mystical expressions of uncertain origin. (One needs to go no further than pondering what is so sacred about the sacral bone.) Without wishing to pass judgment on the plausibility of anyone wanting to address sodium, *exitus* is a noun, meaning exit, and therefore clearly cannot be used to command sodium to exit the cell. The imperative of the verb *exire* (ie, to exit) is *exi* (ie, exit!), while the correct vocative case of *natrium* is *natrium*. “Exit, O sodium!” would therefore be translated as *exi, natrium*. Maintaining a low intracellular sodium concentration may be an imperative for every living cell, but the Latin imperative was obviously not used to denote *ENA*.

To dissect linguistic errors, to which we are admittedly all prone, could be seen as an overzealous pedantry. After all, it is just a wrong word. Has that ever done any harm? Nevertheless, we believe that the case of *exitus natru*, although innocuous in itself, highlights a larger problem. Firstly, although the correct term *exitus natrii* appears in the original 1991 paper on *ENA1* ^15^, the paper continues to be misquoted, which appears to suggest that what is quoted is not necessarily always read. Secondly, *exitus natru*, although incorrect for linguistic reasons, continues to find its way into publications. While bioscientists are not required to use Latin and ancient Greek, nor are they expected to be familiar with the intricacies of their vocabulary and grammar, it is surprising that linguistic errors have not been detected at various stages of the publication process.

That a new term that has drifted away from its original form should take precedence in contemporary literature is hardly surprising. Since the language of life sciences is a dynamic, living entity, it has always been subject to the evolution of existing terms and a constant influx of neologisms. For example, the term *natrium*, for which there is no original ancient equivalent, was first coined by Berzelius in the 1800s.^65^ Moreover, the persistent use of corrupted or incorrect forms often prevails and becomes generally accepted, as an old maxim “*usus (est) tyrannus*” (ie, custom is a tyrant) nicely sums up. *Exitus natru* could therefore be here to stay. In this case, however, it should at least be recognized that *exitus natru* does not stand for the exit (of) sodium in standard Latin. An alternative etymology of *natru*, which we may have inadvertently overlooked, would also be appreciated.

Researchers might perceive ourselves as critical seekers after truth, forming our opinions exclusively on the basis of objective experimental data and their continuous critical (re)appraisal. However, when a word that is not only misquoted but also grammatically incorrect spreads through the scientific literature with such apparent ease, we should pause and ask ourselves whether something similar happens, at least occasionally, not only to the misperceptions of the histories of our research fields but also to the misinterpretations of research data that we all so cherish.

## References

[1] Petersen OH . When a discovery is a rediscovery: do we know the history of our own subject?. Function (Oxf). 2021;2(4):zqab030. 35330619 10.1093/function/zqab030 PMC8788861

[2] Tissot van Patot MC . Getting lost in history: Mabel Purefoy FitzGerald and the origins of hydrochloric acid in the gastric mucosa. Function (Oxf). 2021;2(6):zqab054. 35330791 10.1093/function/zqab054 PMC8788717

[3] Parekh AB, Parekh LBC. “Study the past if you would define the future.”—Confucius. Function (Oxf). 2023;4(1):zqac073. 36686642 10.1093/function/zqac073 PMC9850269

[4] Huxley AF . Discoveries on muscle: observation, theory, and experiment. Br Med J (Clin Res Ed). 1986;293(6539):115–117. 10.1136/bmj.293.6539.115 PMC1340847 3089413

[5] Leslie BR, Gerwin LE, Taylor SI. Sodium–glucose cotransporter-2 inhibitors: lack of a complete history delays diagnosis. Ann Intern Med. 2019;171(6):421–426. 31525753 10.7326/M19-1463

[6] Sugar O . How the sacrum got its name. JAMA. 1987;257(15):2061–2063. 3550163

[7] Scarborough J . Medical Terminologies: Classical Origins. Norman, OK: University of Oklahoma Press; 1992.

[8] Caggiati A, Bergan JJ. The saphenous vein: derivation of its name and its relevant anatomy. J Vasc Surg. 2002;35(1):172–175. 11802151 10.1067/mva.2002.118826

[9] Skou JC . The influence of some cations on an adenosine triphosphatase from peripheral nerves. Biochim Biophys Acta. 1957;23(2):394–401. 13412736 10.1016/0006-3002(57)90343-8

[10] Schatzmann HJ, Witt PN. Action of k-strophanthin on potassium leakage from frog sartorius muscle. J Pharmacol Exp Ther. 1954;112(4):501–508. 13222244

[11] Witt PN, Schatzmann HJ. Effect of strophanthin on potassium liberation in resting and working sartorius muscle in frog. Helv Physiol Pharmacol Acta. 1954;12(2):C44–C46. 13201035

[12] Schatzmann HJ . Cardiac glycosides as inhibitors of active potassium and sodium transport by erythrocyte membrane. Helv Physiol Pharmacol Acta. 1953;11(4):346–354. 13142506

[13] Post RL, Jolly PC. The linkage of sodium, potassium, and ammonium active transport across the human erythrocyte membrane. Biochim Biophys Acta. 1957;25(1):118–128. 13445725 10.1016/0006-3002(57)90426-2

[14] Sen AK, Post RL. Stoichiometry and localization of adenosine triphosphate-dependent sodium and potassium transport in the erythrocyte. J Biol Chem. 1964;239(1):345–352. 14114864

[15] Haro R, Garciadeblas B, Rodriguez-Navarro A. A novel P-type ATPase from yeast involved in sodium transport. FEBS Lett. 1991;291(2):189–191. 1657642 10.1016/0014-5793(91)81280-l

[16] Benito B, Rodriguez-Navarro A. Molecular cloning and characterization of a sodium-pump ATPase of the moss *Physcomitrella patens*. Plant J. 2003;36(3):382–389. 14617094 10.1046/j.1365-313x.2003.01883.x

[17] Wieland J, Nitsche AM, Strayle J, Steiner H, Rudolph HK. The PMR2 gene cluster encodes functionally distinct isoforms of a putative Na^+^ pump in the yeast plasma membrane. EMBO J. 1995;14(16):3870–3882. 7664728 10.1002/j.1460-2075.1995.tb00059.x PMC394466

[18] Benito B, Quintero FJ, Rodriguez-Navarro A. Overexpression of the sodium ATPase of *Saccharomyces cerevisiae*: conditions for phosphorylation from ATP and Pi. Biochim Biophys Acta. 1997;1328(2):214–226. 9315618 10.1016/s0005-2736(97)00098-9

[19] Zahradka J, Sychrova H. Plasma-membrane hyperpolarization diminishes the cation efflux via Nha1 antiporter and Ena ATPase under potassium-limiting conditions. FEMS Yeast Res. 2012;12(4):439–446. 22329368 10.1111/j.1567-1364.2012.00793.x

[20] Deng TC, Yang JY, Sun ML, Zhang YZ, Pan YT, Huang L. Distinct roles of Ena ATP family proteins in sodium accumulation, invasive growth, and full virulence in *Colletotrichum gloeosporioides*. J Fungi (Basel). 2023;9(5):566. 37233277 10.3390/jof9050566 PMC10219246

[21] Markina-Inarrairaegui A, Spielvogel A, Etxebeste O, Ugalde U, Espeso EA. Tolerance to alkaline ambient pH in *Aspergillus nidulans* depends on the activity of ENA proteins. Sci Rep. 2020;10(1):14325. 32868868 10.1038/s41598-020-71297-z PMC7459330

[22] Rodriguez-Navarro A, Benito B. Sodium or potassium efflux ATPase a fungal, bryophyte, and protozoal ATPase. Biochim Biophys Acta. 2010;1798(10):1841–1853. 20650263 10.1016/j.bbamem.2010.07.009

[23] Iizumi K, Mikami Y, Hashimoto M, Nara T, Hara Y, Aoki T. Molecular cloning and characterization of ouabain-insensitive Na(+)-ATPase in the parasitic protist, *Trypanosoma cruzi*. Biochim Biophys Acta. 2006;1758(6):738–746. 16797482 10.1016/j.bbamem.2006.04.025

[24] Esser K, Lemke PA, Bennett JW. The Mycota : A Comprehensive Treatise on Fungi as Experimental Systems for Basic and Applied Research. Berlin, Germany: Springer-Verlag; 1994.

[25] 龚继明, 陈受宜. 离子平衡及其相关信号传导在细胞耐盐中的作用. *中国生物工程 杂 志*. 1999;19(6):2–8.

[26] Adler L . Fungi and salt stress. In: Frankland JC, Magan N, Gadd GM, eds. Fungi and Environmental Change. Cranfield University: Cambridge University Press, 1996:217–234.

[27] Mendoza I, Rubio F, Rodriguez-Navarro A, Pardo JM. The protein phosphatase calcineurin is essential for NaCl tolerance of *Saccharomyces cerevisiae*. J Biol Chem. 1994;269(12):8792–8796. 8132612

[28] Mendizabal I, Rios G, Mulet JM, Serrano R, de Larrinoa IF. Yeast putative transcription factors involved in salt tolerance. FEBS Lett. 1998;425(2):323–328. 9559673 10.1016/s0014-5793(98)00249-x

[29] Lamb TM, Xu W, Diamond A, Mitchell AP. Alkaline response genes of *Saccharomyces cerevisiae* and their relationship to the RIM101 pathway. J Biol Chem. 2001;276(3):1850–1856. 11050096 10.1074/jbc.M008381200

[30] Maresova L, Muend S, Zhang YQ, Sychrova H, Rao R. Membrane hyperpolarization drives cation influx and fungicidal activity of amiodarone. J Biol Chem. 2009;284(5):2795–2802. 19054772 10.1074/jbc.M806693200 PMC2631971

[31] Wong ED, Miyasato SR, Aleksander S et al. Saccharomyces genome database update: server architecture, pan-genome nomenclature, and external resources. Genetics. 2023;224(1):iyac191. 36607068 10.1093/genetics/iyac191 PMC10158836

[32] Dick CF, Meyer-Fernandes JR, Vieyra A. The functioning of Na(+)-ATPases from protozoan parasites: are these pumps targets for antiparasitic drugs?. Cells. 2020;9(10):2225. 33023071 10.3390/cells9102225 PMC7600311

[33] Mackie TD, Brodsky JL. Investigating potassium channels in budding yeast: a genetic sandbox. Genetics. 2018;209(3):637–650. 29967058 10.1534/genetics.118.301026 PMC6028241

[34] Tuteja N, Verma S, Sahoo RK, Raveendar S, Reddy IN. Recent advances in development of marker-free transgenic plants: regulation and biosafety concern. J Biosci. 2012;37(1):167–197. 22357214 10.1007/s12038-012-9187-5

[35] Zajc J, Liu Y, Dai W et al. Genome and transcriptome sequencing of the halophilic fungus *Wallemia ichthyophaga*: haloadaptations present and absent. BMC Genomics. 2013;14(1):617. 24034603 10.1186/1471-2164-14-617 PMC3849046

[36] Sen A, Hsieh WC, Hanna CB et al. The Na(+) pump Ena1 is a yeast epsin-specific cargo requiring its ubiquitylation and phosphorylation sites for internalization. J Cell Sci. 2020;133(16): jcs245415 32694166 10.1242/jcs.245415

[37] Abaza SM . Recent advances in identification of potential drug targets and development of novel drugs in parasitic diseases. Part I: drug resistance. Parasitol United J. 2021;14(3):244–260.

[38] Ruiz A, Arino J. Function and regulation of the *Saccharomyces cerevisiae* ENA sodium ATPase system. Euk Cell. 2007;6(12):2175–2183. 10.1128/EC.00337-07 PMC2168247 17951516

[39] Mou YN, Gao BJ, Ren K, Tong SM, Ying SH, Feng MG. P-type Na(+)/K(+) ATPases essential and nonessential for cellular homeostasis and insect pathogenicity of *Beauveria bassiana*. Virulence. 2020;11(1):1415–1431. 33103596 10.1080/21505594.2020.1836903 PMC7588218

[40] Louis VL, Despons L, Friedrich A et al. *Pichia sorbitophila*, an interspecies yeast hybrid, reveals early steps of genome resolution after polyploidization. G3 (Bethesda). 2012;2(2):299–311. 22384408 10.1534/g3.111.000745 PMC3284337

[41] Son H, Park AR, Lim JY, Lee YW. Fss1 is involved in the regulation of an ENA5 homologue for sodium and lithium tolerance in *Fusarium graminearum*. Environ Microbiol. 2015;17(6):2048–2063. 25627458 10.1111/1462-2920.12757

[42] Arino J, Ramos J, Sychrova H. Alkali metal cation transport and homeostasis in yeasts. Microbiol Mol Biol Rev. 2010;74(1):95–120. 20197501 10.1128/MMBR.00042-09 PMC2832347

[43] Aguilella M, Garciadeblas B, Fernandez Pacios L, Benito B. Phylogenetic and structure-function analyses of ENA ATPases: a case study of the ENA1 protein from the fungus *Neurospora crassa*. Int J Mol Sci. 2023;25(1):514. 38203685 10.3390/ijms25010514 PMC10779151

[44] Illarionov A, Lahtvee PJ, Kumar R. Potassium and sodium salt stress characterization in the yeasts *Saccharomyces cerevisiae, Kluyveromyces marxianus*, and *Rhodotorula toruloides*. Appl Environ Microb. 2021;87(13):e0310020. 10.1128/AEM.03100-20 PMC8315938 33893111

[45] Mahmoud SA . Investigating the mechanisms of action of the SNF2-homolog protein Fun30. Ph.D. thesis. University of Dundee; 2011.

[46] Elicharová H . Homeostáze kationtů alkalických kovů patogenních kvasinek rodu *Candida*. Doctoral thesis. Charles University Faculties; 2017.

[47] Vidal EE . Influência da fonte de nitrogênio no perfil fermentativo, transcriptômico, e na produção de álcoois superiores em *Saccharomyces cerevisiae*. Universidade federal de Pernambuco, Centro de ciências biológicas, Departamento de genética, Programa de pós-graduação em genética; 2012.

[48] Ćosić MV . Efekat apscisinske kiseline u odgovoru odabranih vrsta briofita na Stres izazvan natrijum-hloridom. Ph.D. thesis. Univerzitet u Beogradu Biološki fakultet; 2021.

[49] Peterson MR . Functional characterization of histone deacetylase 2 and histone deacetylase 3 in *Candida albicans*. Ph.D. thesis. University of Kent; 2019.

[50] Kurian SM . Live-cell imaging of the early stages of colony development in *Fusarium oxysporum in vitro* and *ex vivo* during infection of a human corneal model. Ph.D. thesis. University of Manchester; 2016.

[51] Mackie TD . Trafficking and quality control of inward-rectifying potassium channels in the model eukaryote *Saccharomyces cerevisiae*. Doctoral thesis. University of Pittsburgh; 2018.

[52] Tran TTMT . Identifying genes that confer ethanol tolerance in *Saccharomyces cerevisiae*. Ph.D. thesis. The Australian Wine Research Institute, Victoria University; 2011.

[53] David Bosne SCM . Characterization of *Plasmodium falciparum* membrane transporters as potential antimalarial targets. Université Paris-Sud; 2014.

[54] Spillman NJ . Na^+^ regulation in the intraerythrocytic malaria parasite. Ph.D. thesis. Research School of Biology, The Australian National University; 2011.

[55] Plett DC . Spatial and temporal alterations of gene expression in rice. Ph.D. thesis. University of Adelaide, School of Agriculture, Food and Wine; 2008.

[56] Zajc J . Fiziološke in molekularno-biološke prilagoditve halofilne glive wallemia ichthyophaga na življenje v okolju z visokimi koncentracijami soli. Ph.D. thesis. Biotehniška fakulteta, univerzitetni podiplomski študij biomedicine; 2014.

[57] Natali F . Effect of aneuploidy on cellular adaptation to hyper-osmotic and ionic stress. Doctoral thesis. Nanyang Technological University, Singapore; 2022.

[58] Tomassetti S . The transcription factor Sfp1 regulates growth and division in the yeast *Saccharomyces cerevisiae*. Ph.D. thesis; Université de Genève; 2016.

[59] Gerber S . In silico modeling of cation homeostasis in *Saccharomyces cerevisiae*. Dissertation; Mathematisch-Naturwissenschaftlichen Fakultät IHumboldt-Universität zu Berlin; 2011.

[60] Alves DLZ . Fatores de virulência, resistência ao estresse osmótico e susceptibilidade antifúngica de isolados de *Candida tropicalis* oriundos de ambiente costeiro do *Nordeste brasileiro*. Master’s thesis; Universidade federal do Rio Grande do Norte, Centro de ciências saúde, Programa de pós-graduação em ciências farmacêuticas; 2015.

[61] Andršová M . Charakterizace a funkční analýza genu IST2 v kvasince *Saccharomyces cerevisiae*. Master’s thesis; Univerzita Karlova v Praze, Přírodovědecká fakulta Katedra buněčné biologie; 2012.

[62] Rampelotto PH . Molecular Mechanisms of Microbial Evolution. 1st ed. New York, NY: Springer; 2018.

[63] Permiakov EA, Kretsinger RH. Calcium Binding Proteins. Hoboken, NJ: Wiley; 2010.

[64] Churchill WS . A Roving Commission: My Early LIfe. New York, NY: Charles Scribner’s Sons; 1930.

[65] Berzelius JJ . Försök att, genom användandet af den electrokemiska theorien och de kemiska proportionerna grundlägga ett rent vettensk. system för mineralogien. Stockholm, Sweden: A. Gadelius; 1814.

